# Idiopathic Spontaneous Coronary Artery Dissection: A Rare Cause of Acute Myocardial Infarction Successfully Treated With Conservative Management

**DOI:** 10.7759/cureus.106204

**Published:** 2026-03-31

**Authors:** Niko Díaz García, Fernando Carrillo, Elsa Rueda, Carlos Rubén Pérez

**Affiliations:** 1 Internal Medicine, Hospital Santo Tomás, Panama, PAN; 2 Internal Medicine, Ciudad de la Salud, Panama, PAN; 3 Internal Medicine, Hospital Pacifica Salud, Panama, PAN; 4 Cardiology, Hospital Pacifica Salud, Panama, PAN

**Keywords:** case reports, coronary angiography after coronary dissection, coronary vessels, iatrogenic coronary dissection, spontaneous coronary dissection

## Abstract

Spontaneous coronary artery dissection (SCAD) is an uncommon cause of acute coronary syndrome (ACS) characterized by separation of the coronary arterial wall layers, leading to impaired coronary blood flow. It predominantly affects women and has been associated with triggers such as emotional stress and intense physical exertion. We report the case of a 44-year-old woman with no prior cardiovascular history who presented with acute chest pain and elevated troponin levels. Her initial electrocardiogram (ECG) was unremarkable, raising suspicion for non-ST-elevation myocardial infarction (NSTEMI). Subsequent coronary angiography revealed a spontaneous dissection of the first obtuse marginal artery (OM1). During hospitalization, the patient developed atrial fibrillation but remained hemodynamically stable and was managed conservatively. At one-year follow-up, she remained asymptomatic without recurrent angina or arrhythmias. This case highlights the importance of considering SCAD in younger patients, particularly women without traditional cardiovascular risk factors, presenting with symptoms of ACS, even when the initial ECG is non-diagnostic. Early recognition is essential to avoid delays in diagnosis and optimize management.

## Introduction

Spontaneous coronary artery dissection (SCAD) is a non-iatrogenic, non-atherosclerotic condition characterized by separation of the layers of the coronary arterial wall, resulting in the formation of a false lumen that compresses the true lumen and compromises myocardial blood flow [1]. SCAD predominantly affects women, with a reported mean age of 51.8 years, and has an estimated incidence of 0.49% among more than 13 million patients presenting with acute coronary syndrome (ACS) [2,3]. More than half of SCAD cases are associated with underlying conditions such as fibromuscular dysplasia (FMD), recreational drug use, high-dose hormonal therapy, multiparity, Marfan syndrome, and autoimmune disorders. However, approximately 20% of cases are classified as idiopathic [4]. The left anterior descending artery is the most commonly affected vessel, whereas involvement of the obtuse marginal artery is relatively uncommon, accounting for approximately 15% of cases. Here, we present the case of a young woman who presented with SCAD involving the first obtuse marginal artery (OM1) and who was successfully managed with conservative therapy [5].

## Case presentation

A previously healthy 44-year-old female athlete presented to the emergency department with retrosternal chest pain described as oppressive, with an intensity of 7/10. The pain was non-radiating and associated with epigastric discomfort and nausea, persisting for more than six hours. The patient denied recent intense physical exertion, emotional stress, or recent illness. She had no history of hypertension, migraine, connective tissue disease, or prior vascular disorders. She was not pregnant and denied use of hormonal therapy or oral contraceptives. On initial evaluation, her vital signs were: blood pressure 144/86 mmHg (mean arterial pressure 96 mmHg), heart rate 62 beats per minute, respiratory rate 12 breaths per minute, and oxygen saturation 100% on room air. She was alert, oriented, and in no acute distress. Cardiovascular examination revealed regular heart sounds and palpable peripheral pulses. Her Glasgow Coma Scale score was 15/15.

**Figure 1:**
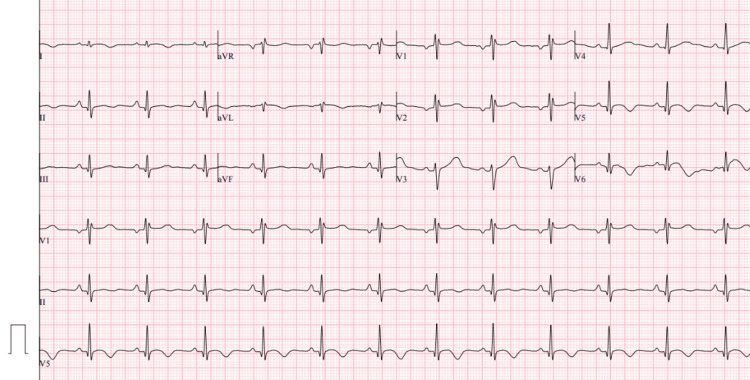
Electrocardiogram on admission Sinus rhythm with findings consistent with lateral subendocardial ischemia, including ST-segment depression in leads V4-V6, I, and aVL.

Laboratory studies, including complete blood count, comprehensive metabolic panel, and renal and liver function tests, were within normal limits (Table 1). Cardiac biomarker analysis revealed elevated high-sensitivity troponin I (0.59 ng/mL; normal value 0.00-0.05 ng/mL) (Table 2). Electrocardiography demonstrated findings consistent with lateral subendocardial ischemia (Figure 1).

**Table 1:** Laboratory studies at admission eGFR, estimated glomerular filtration rate; TSH, thyroid-stimulating hormone; BUN, blood urea nitrogen

Test	Result	Normal values
Leukocytes	9.49x10^3^/uL	4.50-11.50x10^3^/uL
Hemoglobin	14.70 g/dL	12.00-16.00 g/dL
Platelets	236.0x10^3^/uL	150.0-450.0x10^3^/uL
Chloride	104.0 mmol/L	95.0-105.0 mmol/L
Potassium	3.90 mmol/L	3.60-5.00 mmol/L
Glucose	104 mg/dL	70-105 mg/dL
BUN	7 mg/dL	6-20 mg/dL
Alkaline phosphatase	79 U/L	46-122 U/L
Creatinine	0.67 mg/dL	0.50-1.30 mg/dL
eGFR	106.85 mL/min/1.73 m^2^	
Lipase	11 U/L	8-78 U/L
High-sensitivity C-reactive protein	0.65 mg/L	<3.0 mg/L
Aspartate aminotransferase	41 U/L	10-46 U/L
Alanine aminotransferase	14 U/L	9-66 U/L
Total bilirubin	1.10 mg/dL	0.20-1.20 mg/dL
Direct bilirubin	0.40 mg/dL	0.00-0.40 mg/dL
Indirect bilirubin	0.70 mg/dL	0.20-1.20 mg/dL
Total proteins	7.90 g/dL	6.40-8.30 g/dL
Albumin	4.60 g/dL	3.20-5.20 g/dL
Globulin	3.3 g/dL	2.1-3.5 g/dL
TSH	1.9036 uIU/mL	0.3000-5.0000

**Table 2:** Cardiac biomarker results during hospitalization

Test	Day 1	Day 3	Day 5	Normal values
Creatine kinase-myocardial band	23.2 ng/mL	29.7 ng/mL	32.1 ng/mL	0.0-4.3 ng/mL
Myoglobin	162.0 ng/mL	32.3 ng/mL	121.0 ng/mL	0.0-107.0 ng/mL
Troponin I	0.59 ng/mL	4.23 ng/mL	8.88 ng/mL	0.00-0.05 ng/mL
B-type natriuretic peptide	48.9 pg/mL	266.0 pg/mL	211.0 pg/mL	0.0-100.0 pg/mL
D-dimer	<100 ng/mL	125 ng/mL	882 ng/mL	0-600 ng/mL

**Figure 2:**
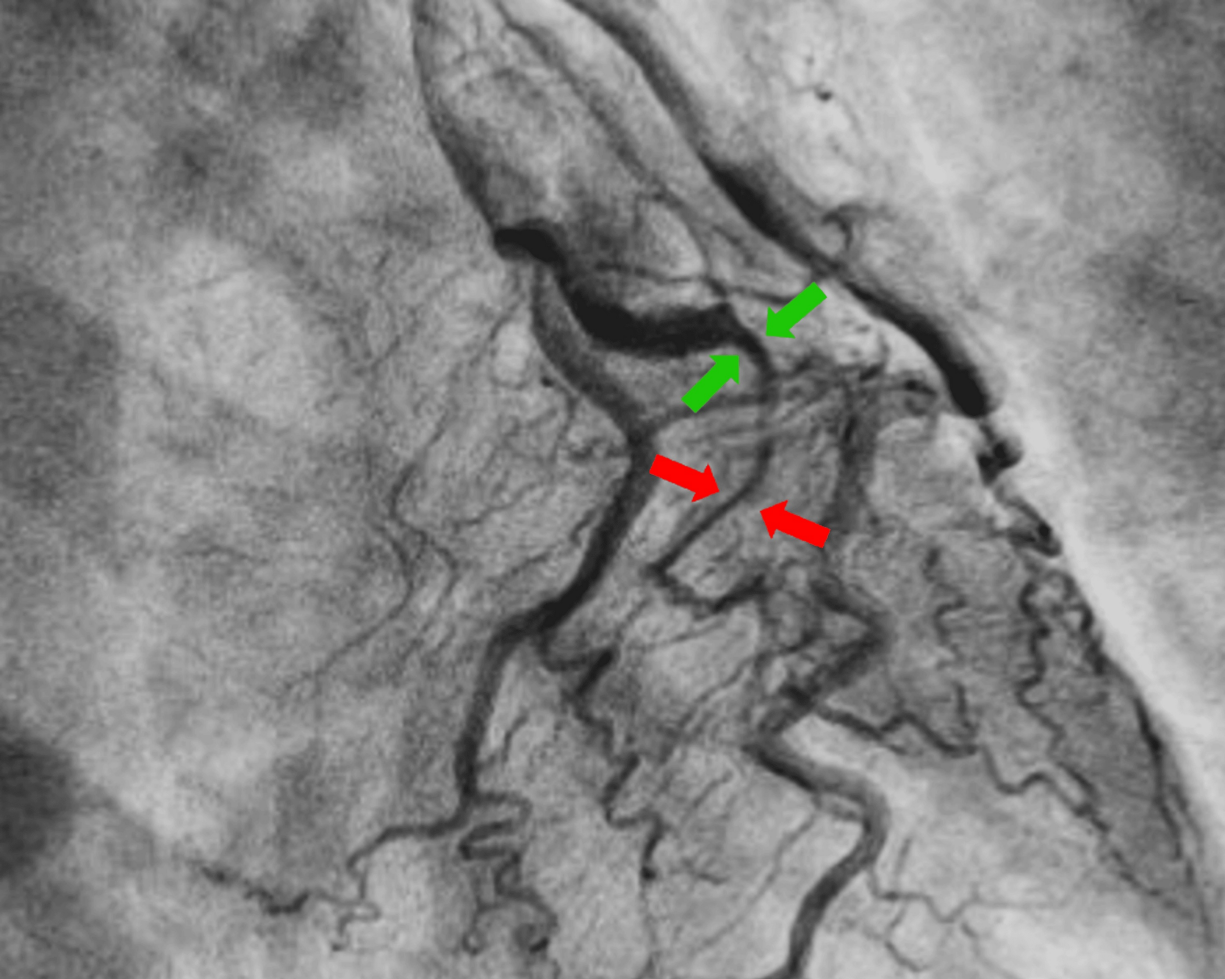
Coronary angiography on admission Angiographic image showing a SCAD involving the mid-to-distal segment of the OM1, characterized by a long segment of smooth, diffuse narrowing consistent with SCAD type 2b (red arrows). A change in vessel caliber is also observed (green arrows). SCAD, spontaneous coronary artery dissection; OM1, first obtuse marginal artery

Given the suspicion of non-ST elevation myocardial infarction, coronary angiography was performed (Figure 2). Based on preserved coronary flow, the patient’s stable condition, the absence of ongoing ischemia, and no left main or proximal vessel involvement, a conservative management strategy was selected.

**Figure 3:**
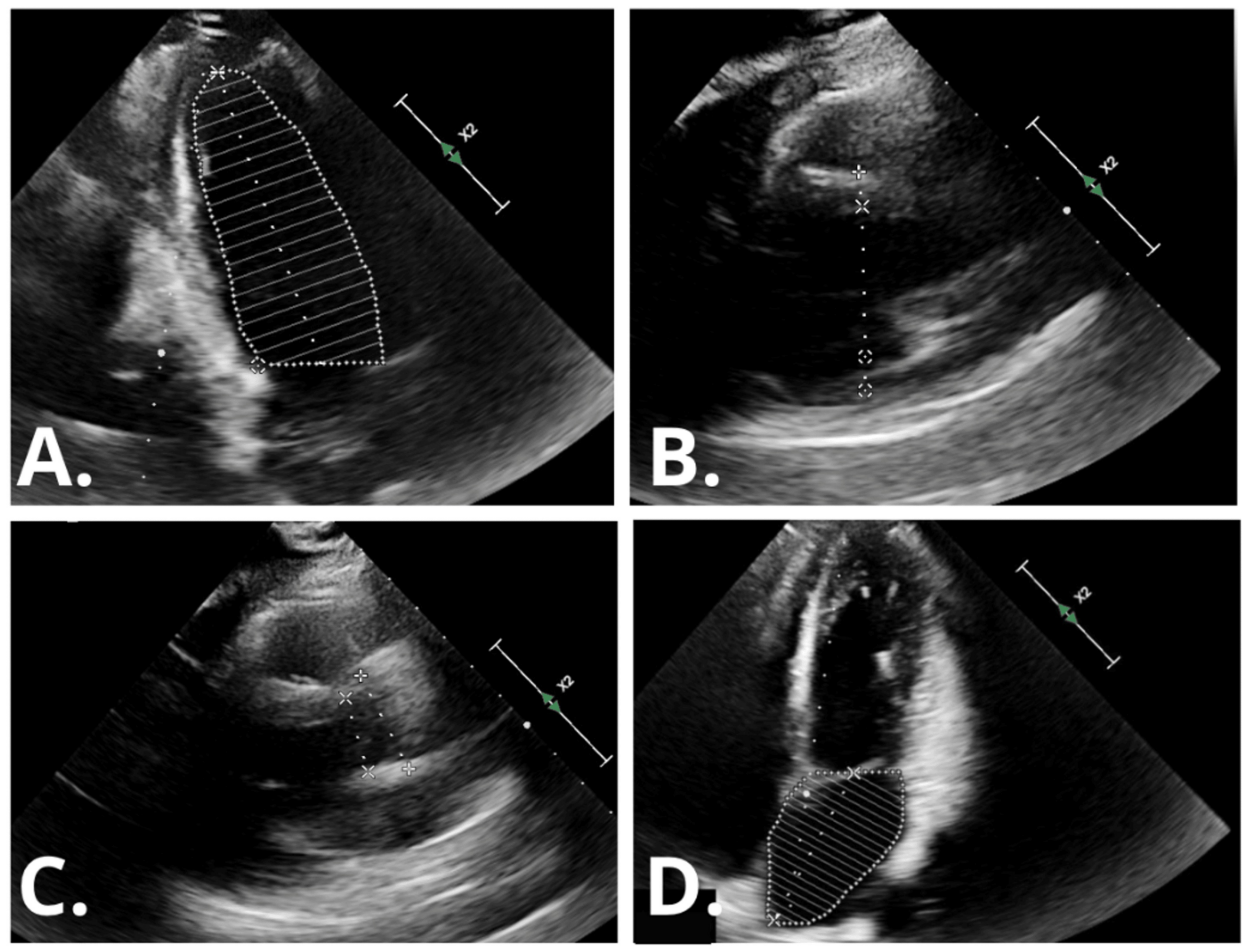
Transthoracic echocardiography findings (A) Apical two-chamber view with endocardial tracing of the left ventricle for volumetric assessment using Simpson’s method. (B) Apical view demonstrating measurement of left ventricular diameter. (C) View showing measurement of the LVOT and aortic root. (D) Apical four-chamber view demonstrating left atrial planimetry. LVOT, left ventricular outflow tract

A transthoracic echocardiogram demonstrated normal left ventricular size and systolic function without regional wall motion abnormalities. The left ventricular ejection fraction was estimated at 71% (Figure 3).

The patient was started on conservative medical therapy consisting of dual antiplatelet therapy, statin therapy, a beta-blocker, and an intravenous nitroglycerin infusion. She was admitted to the intensive care unit for close monitoring.

**Figure 4:**
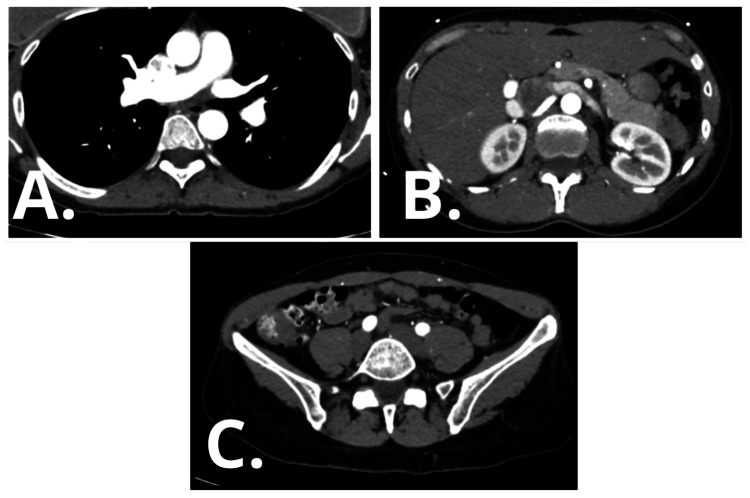
Computed tomography angiography of the thoracic, abdominal, and pelvic vasculature performed to evaluate for associated arteriopathies (A) Thoracic view demonstrating a normal thoracic aorta without aneurysm or dissection. (B) Abdominal view showing normal renal arteries without stenosis or "string-of-beads" appearance suggestive of fibromuscular dysplasia. (C) Pelvic view demonstrating normal iliac arteries. No vascular abnormalities were identified.

To evaluate for associated vascular abnormalities, including FMD, computed tomography angiography of the thoracic, abdominal, and pelvic vasculature was performed. The study demonstrated normal caliber and morphology of the thoracic aorta, abdominal aorta, renal arteries, and iliac arteries, with no evidence of arterial stenosis, aneurysm, dissection, or the characteristic "string-of-beads" appearance suggestive of FMD (Figure 4). Immunologic testing was also unremarkable, effectively excluding vasculitis and other systemic autoimmune conditions (Table 3).

**Table 3:** Immunologic testing *Includes the following IgG autoantibodies: SS-A 60, SS-A 52, SS-B, RNP-70, Sm, RNP/Sm, Scl-70, Centromere B, and Jo-1. ANA, antinuclear antibody; PR3, proteinase 3 antibody; MPO, myeloperoxidase antibody

Test	Result	Normal values
Anti-PR3	2.30 U/mL	Negative <5 U/mL
Anti-MPO	1.00	Negative <5 U/mL
ANA	Negative	Negative <1:160
Anti-DNA	3 U/mL	Negative 25 U/mL
Antinuclear antibody screen*	0.30	Negative <1.0

On the fifth day of hospitalization, the patient developed sudden, severe left shoulder pain accompanied by dyspnea and palpitations. Vital signs at that time were: blood pressure 127/80 mmHg, heart rate 120 beats per minute, respiratory rate 22 breaths per minute, and oxygen saturation 98% on room air. Electrocardiography revealed an irregular rhythm with J-point elevation in leads V3 and V4. The intravenous nitroglycerin infusion was titrated, and treatment with intravenous amiodarone (150 mg bolus followed by continuous infusion) was initiated for paroxysmal atrial fibrillation. A repeat transthoracic echocardiogram demonstrated atrial fibrillation alternating with sinus rhythm, with normal ventricular dimensions and preserved systolic function. No valvular abnormalities or other significant findings were identified.

**Figure 5:**
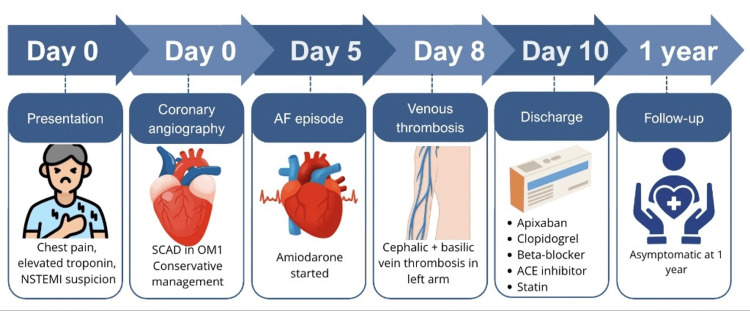
Clinical timeline of the patient’s presentation and follow-up NSTEMI, non-ST-segment elevation myocardial infarction; SCAD, spontaneous coronary artery dissection; OM1, first obtuse marginal artery; ACE, angiotensin-converting enzyme; AF, atrial fibrillation Created by Canva Pro (Canva, Sydney, Australia)

On the eighth day of hospitalization, the patient developed upper extremity superficial venous thrombosis involving the cephalic and basilic veins of the left arm, associated with elevated D-dimer levels (2000 ng/mL) and local inflammatory signs. Given these findings, anticoagulation was initiated (Figure 5).

The patient's condition improved progressively, and she was discharged on the tenth hospital day with prescriptions for apixaban, clopidogrel, bisoprolol, perindopril, and rosuvastatin. Anticoagulation with apixaban was planned for three months. Beta-blocker therapy was continued long-term, given its association with reduced SCAD recurrence. At one-year follow-up, the patient remained clinically stable, with no recurrence of angina or atrial fibrillation.

## Discussion

SCAD is a rare condition, but it is increasingly recognized as an important cause of acute myocardial infarction. It should be considered in patients presenting with ACS accompanied by elevated cardiac biomarkers and ischemic electrocardiographic changes. Chest pain is the most common clinical manifestation, reported in approximately 96% of cases. Less frequently, patients may present with nonspecific symptoms such as nausea, vomiting, or epigastric discomfort, as observed in our patient, which can complicate and delay diagnosis [5].

Several factors have been associated with an increased risk of SCAD. Emotional stress and intense physical exertion are among the most commonly reported precipitating factors, present in more than 50% of cases. Additionally, a strong association exists between SCAD and FMD, which is detected in more than 80% of affected individuals, supporting the recommendation for routine screening for this condition [4]. Other associated disorders include connective tissue diseases such as Marfan syndrome and vascular Ehlers-Danlos syndrome, which are reported in approximately 5% of patients [6].

Electrocardiographic findings in SCAD commonly demonstrate ischemic changes. ST-segment elevation is observed in approximately 46% of cases, while T-wave abnormalities occur in about 22%. However, a normal electrocardiogram does not exclude the diagnosis and may further contribute to diagnostic uncertainty [7,8]. Transthoracic echocardiography (TTE) should be routinely performed to evaluate left ventricular systolic function, identify regional wall motion abnormalities, and exclude alternative diagnoses such as Takotsubo cardiomyopathy [4].

Coronary angiography remains the gold standard for the diagnosis of SCAD. Although the left anterior descending artery is most frequently affected, involvement of the left circumflex artery and its marginal branches, including the obtuse marginal artery, is less common. SCAD most often involves the mid and distal segments of the coronary arteries, while proximal involvement occurs in approximately 10% of cases. According to the angiographic classification, type 2B lesions are characterized by long, diffuse stenosis extending to the distal vessel segment, a pattern observed in our patient [4].

The optimal management strategy for SCAD remains a subject of debate. However, conservative therapy is generally preferred in hemodynamically stable patients with the absence of ongoing ischemia, preserved coronary flow, and lack of left main or proximal vessel involvement.

Medical management commonly includes antiplatelet therapy with aspirin, beta-blockers, and, in selected cases, clopidogrel and statins. Non-pharmacological recommendations focus on minimizing potential triggers, such as intense emotional stress and strenuous physical activity. This conservative approach proved effective in our patient, who remained clinically stable without recurrence [9,10].

## Conclusions

SCAD is an uncommon but important cause of myocardial infarction that can be difficult to recognize due to its variable and sometimes non-specific clinical presentation. It should be considered in younger patients presenting with myocardial infarction, particularly women without traditional cardiovascular risk factors or prior coronary artery disease. Early recognition and an individualized management strategy, often favoring conservative therapy in hemodynamically stable patients, are essential to optimize outcomes and reduce the risk of complications. However, these findings should be interpreted with caution, as they are based on a single case report and may not be generalizable to broader populations.
